# The clinical and cost effectiveness of a Breathlessness Intervention Service for patients with advanced non-malignant disease and their informal carers: mixed findings of a mixed method randomised controlled trial

**DOI:** 10.1186/s13063-016-1304-6

**Published:** 2016-04-04

**Authors:** Morag C. Farquhar, A. Toby Prevost, Paul McCrone, Barbara Brafman-Price, Allison Bentley, Irene J. Higginson, Chris J. Todd, Sara Booth

**Affiliations:** Primary Care Unit, Department of Public Health and Primary Care, University of Cambridge, Institute of Public Health, Robinson Way, Cambridge, CB2 0SR UK; Imperial Clinical Trials Unit, School of Public Health, Imperial College London, Stadium House, 68 Wood Lane, London, W12 7RH UK; Institute of Psychiatry, King’s College London, De Crespigny Park, London, SE5 8AF UK; Formerly of Palliative Care Service, Cambridge University Hospitals’ NHS Foundation Trust, Addenbrooke’s Hospital, Hills Rd, Cambridge, CB2 0QQ UK; Department of Palliative Care, Policy & Rehabilitation, King’s College London, Cicely Saunders Institute, Denmark Hill, London, SE5 9PJ UK; School of Nursing, Midwifery and Social Work, Jean McFarlane Building, University of Manchester, Oxford Rd, Manchester, M13 9PL UK; Department of Oncology, University of Cambridge, Cambridge Biomedical Campus, Cambridge, CB2 0QQ UK

**Keywords:** Breathlessness, Non-malignant disease, Advanced disease, Randomised controlled trial, Complex intervention, Mixed methods, Chronic obstructive pulmonary disease, Palliative care

## Abstract

**Background:**

Breathlessness is the most common and intrusive symptom of advanced non-malignant respiratory and cardiac conditions. The Breathlessness Intervention Service (BIS) is a multi-disciplinary complex intervention, theoretically underpinned by a palliative care approach, utilising evidence-based non-pharmacological and pharmacological interventions to support patients with advanced disease in managing their breathlessness. Having published the effectiveness and cost effectiveness of BIS for patients with advanced cancer and their carers, we sought to establish its effectiveness, and cost effectiveness, in advanced non-malignant conditions.

**Methods:**

This was a single-centre Phase III fast-track single-blind mixed method RCT of BIS versus standard care for breathless patients with non-malignant conditions and their carers. Randomisation was to one of two groups (randomly permuted blocks). Eighty-seven patients referred to BIS were randomised (intervention arm n = 44; control arm n = 43 received BIS after four-week wait); 79 (91 %) completed to key outcome measurement. The primary outcome measure was 0–10 numeric rating scale for patient distress due to breathlessness at four weeks. Secondary outcome measures were Chronic Respiratory Questionnaire, Hospital Anxiety and Depression Scale, Client Service Receipt Inventory, EQ-5D and topic-guided interviews.

**Results:**

Qualitative analyses showed the positive impact of BIS on patients with non-malignant conditions and their carers; quantitative analyses showed a non-significant greater reduction in the primary outcome (‘distress due to breathlessness’), when compared to standard care, of –0.24 (95 % CI: –1.30, 0.82). BIS resulted in extra mean costs of £799, reducing to £100 when outliers were excluded; neither difference was statistically significant. The quantitative findings contrasted with those previously reported for patients with cancer and their carers, which showed BIS to be both clinically and cost effective. For patients with non-malignant conditions there was a notable trend of improvement over both trial arms to the key measurement point; participants may have experienced a therapeutic effect from the research interviews, diluting the intervention’s impact.

**Conclusions:**

BIS had a statistically non-significant effect for patients with non-malignant conditions, and slightly increased service costs, but had a qualitatively positive impact consistent with findings for advanced cancer. Trials of palliative care interventions should consider multiple, mixed method, primary outcomes and ensure that protocols limit potential contaminating therapeutic effects in study designs.

**Trial registration:**

Current Controlled Trials ISRCTN04119516 (December 2008); ClinicalTrials.gov NCT00678405 (May 2008)

**Electronic supplementary material:**

The online version of this article (doi:10.1186/s13063-016-1304-6) contains supplementary material, which is available to authorized users.

## Background

Breathlessness is a disabling and distressing symptom of advanced non-malignant disease that severely reduces the quality of life of patients and their families [1, 2]. Defined as “a subjective experience of breathing discomfort that consists of qualitatively distinct sensations that vary in intensity” [3], it is the main symptom of advanced chronic obstructive pulmonary disease (COPD) but is also the common denominator in many other advanced non-malignant respiratory, cardiac and neuromuscular conditions. In approximately two-thirds of patients presenting with breathlessness the underlying cause is cardiopulmonary disease [4]. Uncontrolled breathlessness can be terrifying for patients and their families: achieving symptom control is therefore a priority [5]. In many instances addressing breathlessness from a symptomatic point of view is the only therapeutic option when it persists in spite of optimised medical management of the underlying condition. However, access to palliative care is known to be poor for patients with advanced non-malignant conditions compared to those with cancer [6–10], despite their well-established need [11, 12].

The Cambridge Breathlessness Intervention Service (BIS) is a multi-disciplinary complex intervention combining non-pharmacological and pharmacological interventions to support breathless patients with any advanced disease in managing their symptom, theoretically underpinned by a palliative care approach [13–15]. Developed and evaluated [16–21] using the Medical Research Council (MRC) framework for complex interventions [22], it has undergone a Phase III RCT with two sub-protocols: one for advanced cancer and one for advanced non-malignant disease (due to differing service models) [23]. The findings of the sub-protocol for patients with advanced cancer have been published previously [21]; this paper reports the findings of the contemporaneously conducted sub-protocol for patients with advanced non-malignant disease.

## Methods

A detailed study protocol, including descriptions of both the intervention and standard care, has been published in an earlier issue of this journal [23]. Subsequent to this the intervention has been more fully described elsewhere [13]. Key aspects of study design, sampling, outcome measures, data collection and analysis for the sub-protocol for patients with advanced non-malignant disease are outlined below. A completed CONSORT 2010 Checklist is provided in Additional file 1.

### Study design

We recruited patients with advanced non-malignant disease referred to BIS into a Phase III mixed method single-blind pragmatic fast-track RCT of BIS versus standard care (July 2008–June 2010). Participants were randomised to one of two groups using randomly permuted blocks of random size 2, 4 and 6, generated by the study statistician and concealed within sealed opaque envelopes until allocation notification by the intervention deliverer: the fast-track (intervention) group received BIS immediately whereas the waiting-list (control) group received BIS after four weeks. All participants continued receiving standard, including palliative, care.

### Participants

Consecutive patients with non-malignant disease who were referred to BIS were invited to participate in the trial, by letter. Patients were eligible if they met BIS referral criteria (they had a diagnosed appropriately treated cause of breathlessness, were troubled by breathlessness in spite of optimisation of underlying illness, and might benefit from a self-management programme) and excluded (from the trial only) if they had received BIS previously. Recruited patients were asked to identify who gave them the most help and support at home (family member or friend); these informal carers were also invited to participate.

### Ethics, consent and permissions

Ethical approval was given by Cambridgeshire2 NHS REC (Ref: 08/H0308/157). All participating patients and carers gave informed consent.

### Participant-reported data and sample size

Patient ‘distress due to breathlessness’ (the primary outcome on which the trial was powered) was measured using a numeric rating scale (NRS). A sample size of 60 randomised patients (26 analysed per arm, with allowance for dropout) would provide 80 % power to detect a 2-point difference in mean distress between groups (SD = 2.5, alpha = 5 %), with increased precision anticipated from adjustment for baseline. Other key patient-reported measures included the Chronic Respiratory Questionnaire (CRQ) [24] and the Hospital Anxiety and Depression Scale (HADS) [25]. Key carer-reported outcome measures included an NRS for carer distress due to patient breathlessness and the HADS. The EQ-5D [26] and Client Service Receipt Inventory (CSRI) [27] were administered for the health economic analyses. Brief qualitative topic-guided interviews were also conducted with all patients and carers to explore their expectations and experiences of BIS. A full list of baseline measures and outcomes is reported in the published protocol [23].

### Data collection

Participating patients completed a baseline interview (t1) prior to randomisation. This mixed method interview included both the quantitative patient-reported measures and the brief qualitative topic-guided interview described above. Carers were interviewed separately where possible, using a mixed method interview of quantitative carer-reported measures and a brief qualitative topic-guided interview. Similar mixed method follow-up interviews were conducted with both patients and carers at each subsequent follow-up interview (t2–t5) at fortnightly intervals. Interview two (t2; two weeks after baseline) was designed to represent the midway point in either receiving the BIS intervention for the intervention arm or the waiting-list period for the controls; interview three (t3) was designed to represent completion of BIS for the intervention arm, or the end of the waiting-list period prior to commencing BIS for the controls; interview four (t4) was designed to represent the midway point in receiving the BIS intervention for the controls (no t4 was conducted with patients and carers on the intervention arm); and the final interview (t5; eight weeks from baseline) was designed to represent four weeks after BIS for the intervention arm, and the completion of BIS for the controls. Data collection was designed to facilitate researcher blinding to group allocation for the collection of primary and key secondary outcomes at t3. That is, researcher blinding was explained to study participants on recruitment, they were reminded at the start of t2 not to let the researcher know their group allocation, and at the start of t3 they were asked not to let the researcher know their group allocation until the researcher came to open their group allocation envelope just prior to CSRI completion (after the collection of primary and key secondary outcomes at t3). Both clinical and administrative staff were also reminded of the importance of researcher blinding throughout the study.

### Analysis

Intention-to-treat analyses, within completers, were conducted using a linear regression model; each outcome was adjusted for its baseline. Costs were calculated by combining service use data (collected for the two months prior to baseline and at four-week follow-up) with UK 2011/12 unit costs [28]. The cost of the intervention was calculated at £91 per contact based on specialist nursing input costs, with phone contacts costed at 25 % of this. Costs were combined with the primary outcome and EQ-5D-derived quality-adjusted life years (QALYs), with uncertainty explored using cost-effectiveness planes.

Qualitative interview data were transcribed and anonymised. As first described in our paper reporting the results of the sub-protocol for patients with cancer [21], two approaches were taken to the analysis of this unusually comprehensive qualitative dataset. First, transcripts of t3 interviews from all patients and carers were categorised into one of three intervention impact levels by three analysts working independently (Level 1: Significant impact - clearly stated BIS made a difference; Level 2: Some impact - no major change recognised, but valued specific aspects of BIS; Level 3: No impact – BIS made no difference at all). Categorisation commenced with a small number of interviews. The three analysts then met to compare their categorisations, discussing and resolving differences through clarifying both level definitions and data interpretation, before repeating this process for all remaining interviews with patients with non-malignant disease and their carers.

Second, as qualitative analysis of this size of dataset would be unmanageable, 20 patient (and associated carer) intervention arm transcripts were purposefully sampled against a four-cell matrix of t3 changes in the primary outcome, to achieve a maximum diversity sample [29]. The four cells represented: (Cell 1) patients who improved most on the primary outcome (who, predictably, had high baseline scores; the Biggest Improvers); (Cell 2) patients with high baseline scores (to match Cell 1) but who improved least (Limited Improvers); (Cell 3) patients who worsened (who transpired to have low-middling baseline scores; Worseners); and (Cell 4) patients with closest matches to Cell 3 baseline scores but who improved most (Moderate Improvers). Anonymised interview transcripts for this purposive sample were imported into NVivo software [30] to facilitate framework analysis [31]. This analysis explored patient and carer reports of the nature of the impact of BIS and which aspects of BIS were valued, and sought to identify possible mechanisms of impact.

## Results

### Recruitment and baseline characteristics

**Fig. 1:**
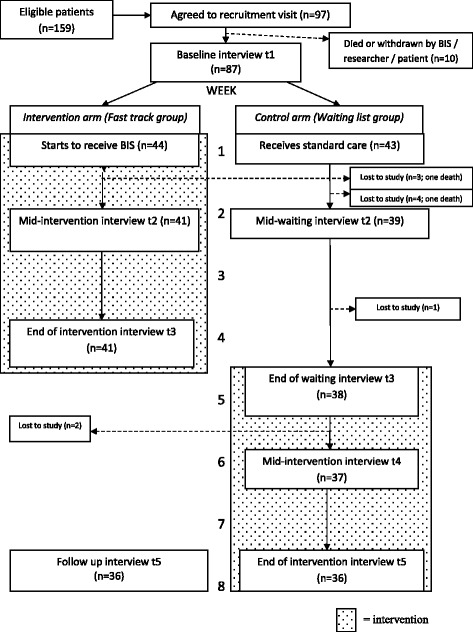
CONSORT flow diagram

Figure 1 (CONSORT flow diagram) indicates that we randomised 87 patients and 79 (91 %) completed the trial to the key outcome measurement (t3). Eight patients withdrew from the trial prior to t3: three and five from the intervention and control arms respectively (including one death in each arm). Researchers remained blinded to group allocation for 67 % (53/79) of patients for collection of the t3 primary outcome.

Baseline characteristics were well matched across trial arms (Tables 1 and 2). Patients were predominantly elderly, male and living with others. COPD was the commonest non-malignant disease (predominantly GOLD stage 3 or 4; severe to very severe). Their mean HADS anxiety score was slightly higher than population norms and their mean HADS depression score notably higher (mean population norm for anxiety 6.14 (SD = 3.76) and depression 3.68 (SD = 3.07) [32]). Just over half (n = 46, 53 %) had anxiety scores that were clinically meaningful (clinically meaningful threshold of 7) and just under half (n = 39; 45 %) had clinically meaningful depression scores. Breathlessness, performance status and co-morbidity were as anticipated for these patients. Patient ‘distress due to breathlessness’ and CRQ domain scores were similar across trial arms.

**Table 1:** Baseline characteristics by arm, BIS Phase IIInm - patients with non-malignant conditions

Baseline characteristics - patients	Mean (SD) or % (n)		
	Intervention arm (fast track)	Control arm (waiting list)	Total
Age (years)	72.3 (10.6)	72.2 (9.4)	72.2 (9.9)
Sex (male)	64 % (28)	58 % (25)	61 % (53)
Lives alone	21 % (9)	37 % (16)	29 % (25)
Diagnosis:			
COPD Other non-malignant	80 % (35) 20 % (9)	88 % (38) 12 % (5)	83 % (72) 17 % (14)
GOLD COPD classification (if available):			
1 - mild 2 - moderate 3 - severe 4 - very severe	9 % (2) 9 % (2) 48 % (11) 35 % (8) (23/35 with COPD)	0 % (0) 32 % (7) 36 % (8) 32 % (7) (22/38 with COPD)	5 % (2) 20 % (9) 42 % (19) 33 % (15) (45/72 with COPD)
Charlson Co-Morbidity Index (0-9)^a^	4.4 (1.39)	4.4 (1.46)	4.4 (1.42)
Australia-modified Karnofsky Performance Scale (0-100)^b,c^	66.8 (14.9)	65.8 (14.9)	66.3 (14.9)
Modified Borg at rest (0-10)^a^	1.9 (1.27)	1.9 (1.05)	1.9 (1.16)
Modified Borg on exertion (0-10)^a^	6.5 (2.28)	6.0 (2.18)	6.2 (2.23)
NRS worst breathlessness 24 h (0-10)^a^	6.0 (2.14)	5.6 (2.29)	5.8 (2.21)
NRS breathlessness now (0-10)^a^	2.8 (2.13)	2.8 (1.91)	2.8 (2.01)
NRS average breathlessness 24 h (0-10)^a^	4.5 (1.99)	4.7 (1.83)	4.6 (1.91)
NRS distress due to breathlessness (0-10)^a^	6.2 (2.50)	5.9 (3.17)	6.0 (2.84)
Anxiety score (HADS; 0-21)^a^	7.84 (3.79)	8.86 (4.75)	8.35 (4.31)
Depression score (HADS; 0-21)^a^	6.81 (3.27)	7.84 (3.72)	7.33 (3.52)
Anxiety (HADS)^a^:			
Normal (0-7) Mild (8-10) = possible clinical disorder Moderate (11-14) = probable clinical disorder Severe (15-21) = probable clinical disorder	42 % (18) 35 % (15) 18 % (8) 5 % (2)	51 % (22) 9 % (4) 26 % (11) 14 % (6)	47 % (40) 22 % (19) 22 % (19) 9 % (8)
Depression (HADS)^a^:			
Normal (0-7) Mild (8-10) = possible clinical disorder Moderate (11-14) = probable clinical disorder Severe (15-21) = probable clinical disorder	58 % (25) 28 % (12) 12 % (5) 2 % (1)	51 % (22) 17 % (7) 30 % (13) 2 % (1)	55 % (47) 22 % (19) 21 % (18) 2 % (2)
CRQ dyspnoea score (1-7)^b^	3.12 (0.91)	3.06 (0.92)	3.09 (0.91)
CRQ fatigue score (1-7)^b^	3.15 (0.96)	2.76 (1.18)	2.95 (1.09)
CRQ emotional function score (1-7)^b^	3.95 (1.05)	3.78 (1.18)	3.86 (1.12)
CRQ mastery score (1-7)^b^	3.87 (1.28)	3.91 (1.34)	3.89 (1.30)
Number of respondents	37-44	37-43	74-87

^a^High score is worse

^b^High score is better

^c^A score of 60 represents ’Requires occasional assistance but is able to care for most of needs’

**Table 2:** Baseline characteristics by arm, BIS Phase IIInm – informal carers of patients with non-malignant conditions

Baseline characteristics - carers	Mean (SD) or % (n)		
	Intervention arm (fast track)	Control arm (waiting list)	Total
Carer age (years)	62.5 (14.82)	62.0 (12.02)	62.2 (13.39)
Carer sex (female)	79 % (23)	79 % (22)	79 % (45)
Carer employment status:			
Employed – full time Employed – part time Voluntary work Retired Other (e.g., unemployed due to illness/student)	22 % (6) 22 % (6) 0 % (0) 48 % (13) 8 % (2)	8 % (2) 4 % (1) 4 % (1) 73 % (19) 11 % (3)	15 % (8) 13 % (7) 2 % (1) 60 % (32) 10 % (5)
NRS carer distress due to patient’s breathlessness (0-10)^a^	5.0 (2.80)	4.5 (2.75)	4.7 (2.76)
Carer anxiety score (HADS; 0-21)^a^	7.64 (4.97)	7.69 (4.36)	7.67 (4.64)
Carer depression score (HADS; 0-21)^a^	5.04 (3.89)	5.04 (3.83)	5.04 (3.82)
Carer anxiety (HADS)^a^:			
Normal (0-7) Mild (8-10) = possible clinical disorder Moderate (11-14) = probable clinical disorder Severe (15-21) = probable clinical disorder	52 % (13) 28 % (7) 8 % (2) 12 % (3)	44 % (10) 30 % (7) 17 % (4) 9 % (2)	48 % (23) 29 % (14) 13 % (6) 10 % (5)
Carer depression (HADS)^a^:			
Normal (0-7) Mild (8-10) = possible clinical disorder Moderate (11-14) = probable clinical disorder Severe (15-21) = probable clinical disorder	84 % (21) 4 % (1) 8 % (2) 4 % (1)	74 % (17) 13 % (3) 13 % (3) 0 % (0)	79 % (38) 8 % (4) 10 % (5) 2 % (1)
Number of respondents	24-29	21-28	45-57
No carer interview	14	15	29

^a^High score is worse

Carers were predominantly older women, and about half were in employment. As with the patients their mean HADS anxiety and depression scores were higher than population norms [32]. Anxiety scores were clinically meaningful for just over half of the carers (n = 25, 52 %) and just under a quarter (n = 10, 21 %) had clinically meaningful depression scores. Carer ‘distress due to patient breathlessness’ was lower than patient distress, but similar across trial arms.

### Change in patient and carer distress due to breathlessness

Comparison of change in patient distress due to breathlessness (primary outcome measure; NRS range 0–10) from baseline (t1) to the key outcome measurement point (t3) showed that patients randomised to the intervention arm achieved a greater, 2.22-point, reduction in their distress due to breathlessness compared with a 1.56-point reduction for controls; however, this was not statistically significant: adjusted difference of –0.24 (95 % CI: –1.30 to 0.82), *p* = 0.65 (Table 3). Carers of patients randomised to the intervention arm achieved a greater, 1.03-point, reduction in their distress due to their patient’s breathlessness compared with a 0.2-point increase for controls, but again this was not statistically significant: adjusted difference of –0.42 (95 % CI: –1.86 to 1.02), *p* = 0.56 (Table 4).

**Table 3:** Changes in patient distress due to breathlessness (primary outcome), mastery, anxiety and depression, by trial arm, for patients with non-malignant conditions

	t1^a^	t2	t3	t4	t5	Difference in mean t3 adjusted for baseline (I minus C)	With 95 % confidence interval	*p*-value
	Mean (SD)	Mean (SD)	Mean (SD)	Mean (SD)	Mean (SD)			
Control arm (waiting list)	Baseline	Control	Completed control	Midway intervention	Completed intervention			
Intervention arm (fast-track)	Baseline	Midway intervention	Completed intervention	Post-intervention	Post-intervention			
Primary outcome: NRS distress due to breathlessness (0-10)^b^								
Control arm (waiting list)	5.61 (3.23)	5.03 (2.84)	4.05 (2.57)	3.73 (2.85)	3.36 (2.63)	-0.24	(-1.30,0.82)	*p* = 0.65
Intervention arm (fast-track)	6.24 (2.53)	4.66 (2.85)	4.02 (2.49)	n/a	4.25 (2.92)			
Key secondary outcomes:								
CRQ^c^ mastery (1-7)								
Control arm (waiting list)	4.13 (1.25)	4.03 (1.25)	4.24 (1.17)	4.42 (1.30)	4.74 (1.09)	0.43	(-0.02,0.89)	*p* = 0.06
Intervention arm (fast-track)	3.85 (1.33)	4.44 (1.29)	4.49 (1.35)	n/a	4.72 (1.11)			
HADS^b^ anxiety (0-21)								
Control arm (waiting list)	8.32 (4.30)	9.05 (4.47)	8.61 (4.25)	8.00 (4.83)	7.56 (4.16)	-0.76	(-1.95,0.44)	*p* = 0.21
Intervention arm (fast-track)	7.80 (3.87)	7.77 (3.49)	7.45 (3.97)	n/a	7.57 (3.82)			
HADS^b^ depression (0-21)								
Control arm (waiting list)	7.71 (3.81)	7.97 (4.11)	7.71 (3.83)	7.86 (4.67)	7.47 (4.16)	-0.61	(-1.76,0.54)	*p* = 0.29
Intervention arm (fast-track)	6.73 (3.32)	6.62 (3.65)	6.28 (3.97)	n/a	6.80 (4.21)			
Number of respondents	77-79	75-80	77-79	35-37	69-72			

^a^For those with a t3 score

^b^High score is worse

^c^High score is better

**Table 4:** Changes in carer distress due to their patient’s breathlessness and carer anxiety, by trial arm, for carers of patients with non-malignant conditions

	t1^a^	t2	t3	t4	t5	Difference in mean t3 adjusted for baseline (I minus C)	With 95 % confidence interval	*p*-value
	Mean (SD)	Mean (SD)	Mean (SD)	Mean (SD)	Mean (SD)			
Control arm (waiting list)	Baseline	Control	Completed control	Midway intervention	Completed intervention			
Intervention arm (fast-track)	Baseline	Midway intervention	Completed intervention	Post-intervention	Post-intervention			
Carer NRS distress due to patient’s breathlessness (0-10)^b^								
Control arm (waiting list)	4.24 (2.72)	4.48 (3.06)	4.44 (3.14)	3.67 (2.88)	4.04 (3.21)	-0.42	(-1.86,1.02)	*p* = 0.56
Intervention arm (fast-track)	5.30 (2.67)	4.22 (2.92)	4.27 (2.48)	n/a	4.25 (2.99)			
Key secondary outcomes:								
Carer HADS^b^ anxiety (0-21)								
Control arm (waiting list)	7.55 (4.54)	7.74 (5.22)	7.40 (5.24)	7.42 (6.34)	7.08 (5.92)	-1.22	(-2.84,0.40)	*p* = 0.14
Intervention arm (fast-track)	7.65 (5.19)	7.00 (4.69)	6.00 (4.29)	n/a	6.90 (5.08)			
No. of respondents	44	46	51	24	45			

^a^For those with a t3 score

^b^High score is worse

### Change in patient mastery of breathlessness, and patient and carer anxiety and depression

Mean CRQ mastery scores improved slightly by t3 on both arms with greater improvement in the intervention arm; not statistically significant (Table 3). No significant differences were found between trial arms to t3 on other CRQ domains (dyspnoea, fatigue or emotional function). Mean patient anxiety scores decreased slightly for the intervention arm and increased slightly for the control arm by t3 and mean depression scores decreased slightly by t3 in the intervention arm and remained stable for controls; none of these modest changes was statistically significant (Table 3). Mean anxiety scores for carers achieved a greater, 1.65-point, reduction by t3 in the intervention arm compared with a 0.15-point reduction for controls, but again this was not statistically significant: adjusted difference of –1.22 (95 % CI: –2.84 to 0.40), *p* = 0.14 (Table 4). There was little change in other patient or carer secondary outcomes.

### Reported benefit of BIS

Categorisation of qualitative interviews (n = 78; no qualitative interview for one patient) indicated that 56 % (n = 44) of patients, or patient-carer dyads, inferred that BIS had had a significant impact (Level 1). A further 36 % (n = 28) suggested BIS had had some impact (that is, no major change noted, but they valued specific aspects of BIS; Level 2) and 8 % (n = 6) reported no impact (Level 3) (Table 5).

**Table 5:** Reported benefit of BIS for patients with non-malignant conditions

Results of categorisation of t3 (intervention arm: fast track) and t5 (control arm: waiting list) patient and carer qualitative interviews into levels of impact (Impact Categorisation Levels)		
Level 1: Significant impact – clearly stated BIS made a difference	Level 2: Some impact – no major change recognised, but valued specific aspects of BIS	Level 3: No impact – BIS made no difference at all
56 % (44/78)	36 % (28/78)	8 % (6/78)

(no qualitative interview for one patient)

Table 6 shows the Impact Categorisation Levels for the purposively sampled qualitative interviews (sampled for maximum diversity of change on primary outcome to t3). As in the sub-protocol for patients with cancer, given the skewed distribution of the Impact Categorisation Levels it is unsurprising that most were Levels 1 and 2, even among ‘Worseners’ on the primary outcome (Cell 3).

**Table 6:** Purposively sampled t3 qualitative interviews with patients with non-malignant conditions (intervention arm) and their Impact Categorisation Levels

Change in NRS distress due to breathlessness^a^ (primary outcome measure) from t1 to t3 (and Impact Categorisation Level)	
Cell 1: Biggest Improvers (from high baseline scores)	Cell 2: Limited Improvers (high baseline score Cell 1 matches who improved the least)
068: NRS distress reduced from 8-2 (Level 1)	002: NRS distress unchanged at 8-8 (Level 2)
012: NRS distress reduced from 9-3 (Level 1)	038: NRS distress reduced from 8-6 (Level 1)
137: NRS distress reduced from 10-3 (Level 1)	059: NRS distress reduced from 10-7 (Level 2)
140: NRS distress reduced from 10-2 (Level 1)	103: NRS distress reduced from 10-9 (Level 1)
100: NRS distress reduced from 8-0 (Level 1)	109: NRS distress reduced from 9-7 (Level 3)
Cell 3: Worseners (who turned out to have a low-middling baseline scores)	Cell 4: Moderate Improvers (closest baseline score Cell 3 matches who improved the most)
072: NRS distress increased from 5-8 (Level 1)	015: NRS distress reduced from 5-2 (Level 3)
084: NRS distress increased from 6-9 (Level 2)	036: NRS distress reduced from 5-0 (Level 2)
126: NRS distress increased from 5-7 (Level 1)	042: NRS distress reduced from 6-2 (Level 1)
158: NRS distress increased from 1-3 (Level 1)	161: NRS distress reduced from 5-0 (Level 2)
027: NRS distress increased from 3-4 (Level 2)	108: NRS distress reduced from 6-1 (Level 3)

^a^High score is worse

Level 1 = significant impact; Level 2 = some impact; Level 3 = no impact.

Numbers to the left = study identity numbers

Qualitative analysis of the purposively sampled interviews identified the nature of the impacts of BIS and which aspects of the BIS model were valued, and also sought to identify possible mechanisms of impact. The findings of these qualitative analyses for patients with non-malignant conditions and their carers reaffirmed the findings for patients with cancer and their carers reported previously [21]. Patients with non-malignant conditions and their carers described a range of impacts including reduced fear, anxiety, worry, and feelings of panic (“*with the breathlessness I wasn’t scared […] I did a lot [of activity] yesterday and still I wasn’t panicked or distressed*” [158t3p]), as well as feeling more confident about breathlessness. They valued the multi-disciplinary staff expertise (their knowledge and understanding of life with breathlessness), the characteristics of the BIS staff (their approachability and attentiveness) and their reassuring and positive approach, and the time BIS gave them to talk about breathlessness with an expert (“*This was somebody who knew what we were talking about, talked the same language*” [042t3p]). Like the patients with cancer [21], they again reported that being seen at home was especially helpful.

Patients with non-malignant conditions and their carers identified the same BIS-delivered interventions they found therapeutic as reported previously for patients with cancer and their carers [21]. These included: providing a handheld fan and teaching patients how to use it; encouragement of exercise and goal-setting; coaching in breathing techniques, positioning, pacing and relaxation; providing occupational therapy aids; information and education — again, as reported for patients with cancer [21], learning that “being breathless won’t kill me” was particularly important to some patients and carers. Again patients with non-malignant conditions reported the benefits of advice on daily strategies to ease breathlessness, and again many referred to these as “lots of little tips”, but patients with non-malignant conditions and their carers particularly valued how the service reviewed their established practices and strategies, praised effective approaches, and provided reassurance. As with the patients with cancer [21], our explanatory analysis suggests that it was not only the provision of these interventions that was important, but also that *how* they were delivered was key to their impact: delivery of interventions through the provision of knowledge (why and how interventions work or specific guidance on how and when to use a particular intervention) increased patients’ and carers’ confidence and legitimised strategies that at first appeared too simple to be effective or to have much impact. Thus, as for patients with cancer and their carers, the mechanism of impact of BIS appears to relate to the acknowledgement and validation of breathlessness, and improved knowledge about breathlessness, which enhances patients’ and carers’ understanding and their confidence in living with the symptom, reducing feelings of being alone with breathlessness. Table 7 provides illustrative quotes for the gaining of knowledge and confidence, and some participant-identified interventions for patients with non-malignant conditions and their carers.

**Table 7:** Illustrative quotes about mechanisms of impact and valued interventions from purposive sample of patients with non-malignant conditions

Mechanisms of impact
Mechanism of impact - gaining knowledge: Patient: “Well I’ve gone back to my choir last Friday, first time in 6 months … and it was fantastic, I was so happy to be there, yeah, really pleased to be there. Couldn’t do the singing as much as I would like to, but it’s coming, it’s coming. And it was lovely because everybody was pleased to see me and lots of hugs and kisses, so … it was really nice […] I think it is the Breathlessness Service has done it […] talking it over with experts, having people come to the house giving me pointers of how I can improve my daily living” [137t3p; Impact Categorisation Level 1 – Significant impact; Cell 1 – Biggest Improvers on primary outcome]
Mechanism of impact - feeling not alone: Carer: “the fact that there are things out there […] it is the reassurance and support really […] we have felt we’ve had support from everybody, and I’m always telling people […] what marvellous support we’ve had, and it does make a difference. You know the outcome’s not going to be really any different, but it does make a difference to have that support, definitely” [012t3pc; Impact Categorisation Level 1 – Significant impact; Cell 1 – Biggest Improvers on primary outcome]
Mechanism of impact - gaining confidence: Patient: “the next thing she said is cool air, you know, plenty of air, and gave me that fan, and that when you are sort of out of breath […] use that for about 10 minutes, and she showed me how to breathe in and went not ‘ha’ *(harsh)* like that but ‘ha’ *(soft)* like that, you know, and she explained all that to me, and then the next thing she said is relaxing, not tensed up, and how I should position myself, sitting, lying down, and so on, you know, everything that … even standing, how I should do it, and she gave me the notes on it […] it gave me a lot of confidence with myself, which I didn’t have before, with this breathing […] it gave me a lot of confidence in the sense that I’m more relaxed about breathing, and even smoke less” [158t3p; Impact Categorisation Level 1 – Significant impact; Cell 3 – Worsener on primary outcome]
Valued interventions
Valued intervention - handheld fan: Patient: “She said this [fan] might help instead of the oxygen. […] When I’m just a little bit out of breath or first thing in the morning… when I’m coughing and spluttering I start getting short of breath, I can lay in bed and use that, so I don’t have oxygen upstairs. […] I take it to work [and] I can get out of the lorry more […] because I know […] I’ll put my hand in my pocket, turn it on as I’m walking back to my cab *(puts fan on)* and by the time I get to the cab I’m OK. Before I had it I used to have to stand at the lorry door and catch my breath […] when I’ve got a chest infection, like now, I get to the back of the lorry [and] the weather’s wrong or I grab a bin wrong… ‘phew’ [but] I can put this on, walk back to the cab. Whereas before I had this like… if I had a chest infection I’d stay on my arse all day” [126t3p; Impact Categorisation Level 1 – Significant impact; Cell 3 – Worsener on primary outcome]
Valued intervention - positioning: Patient: “well it was her who told me about my shoulders, which was very helpful I thought […] you get a pillow under each one […] and you relax your shoulders […] I found that very helpful actually, you know, I think ‘well, I’ve got to do my shoulders’ […] That’s entirely new to me that was. I’m surprised the exercise places I’ve been [didn’t mention] the shoulders. Amazing that is, absolutely amazing […] She said it puts a lot of strain on your shoulders by keeping them up all the time, you know, and she said do that […] and she showed me about the pillows, and […] that makes a difference” [036t3p; Impact Categorisation Level 2 – Significant impact; Cell 4 – Moderate Improver on primary outcome]
Valued intervention – “breathlessness won’t kill you”: Carer: “when he’s breathless he panics naturally because he’s always felt that […] he was going to die, but [the BIS doctor] said ‘that will not happen, not in one of your breathless attacks, you will not die in an attack’, which helped me because you know, I then have to think ‘oh my gosh, what can I do to help him’…you know. So she did give me some leaders as to what I can do to help, knowing now that he won’t die in one of these sort of situations, so that certainly helped me, and it certainly helped me to realise that, you know, I can probably help him to calm down. So yes, as a carer I think it was a help.” [038t3c; Impact Categorisation Level 1 – Significant impact; Cell 2 – Limited Improvers on primary outcome]

As with patients with cancer [21], reviewing transcripts for the categorisation of impact levels exercise identified BIS contacts beyond the key measurement point for the primary outcome for a substantial proportion of patients with non-malignant conditions: 39 % of patients (30/78) described planned BIS contacts beyond t3. Thus, as was noted for with patients with cancer [21], there may have been further benefit beyond the primary outcome at t3.

In addition, this review of transcripts identified the potential therapeutic effect of the research interviews on patients with non-malignant conditions and their carers as well as the difficulty some had in separating their researcher from the intervention. Nearly a quarter of patients with non-malignant conditions and/or their carers explicitly stated that talking to their researcher about breathlessness was helpful: 23 % (18/78) compared with 18 % of patients with cancer and their carers (10/54). Just over a quarter had difficulty separating their researcher from the intervention: 26 % (20/78) compared with 15 % of patients with cancer and their carers (8/54). Table 8 provides illustrative quotes for the potential therapeutic effect of the research interviews on patients with non-malignant conditions and their carers.

**Table 8:** Illustrative quotes on the potential therapeutic effect of research interviews for patients with non-malignant conditions and their carers

Interviewer: “[…] what did you find helpful?” Carer: “I think being able to talk to somebody other than… a total stranger shall we say… and like yourself, I mean I feel as if I’ve known you for years, it’s strange […] you know, you feel like part and parcel of the family” [Carer 108t3c]
Patient: “[being] able to talk to somebody who understands. Because, with the best will in the world, people who don’t have breathing problems don’t understand what it’s like not to be able to breathe. All of them, my family, friends, everybody, because you all do it automatically, you don’t have to think about it, they’ve got no idea what it’s like, but talking to people who do understand like yourself, like [BIS team member], like the doctors, is helpful […] talking it over […] because you and [BIS team member] are approachable […] I feel as though I could talk to you, and I felt I could talk to her, I didn’t feel intimidated” [Patient 137t3p]
Patient: “being able to talk to different people, yourself included, you realise that you can cope, and if you do what you’re told sort of thing… I suppose like a child! […] I’m glad that I’ve spoken to you, yourselves, and other people, and I don’t find any fault with any of you at all” [Patient 108t3p]

### Costs and cost effectiveness

In the two months prior to baseline the waiting-list group was more likely to have had inpatient care, and this resulted in a difference in total costs of £1,678 (Table 9). Other service use at baseline was similar between the two groups. The difference in inpatient costs was in the opposite direction during the follow-up period and total costs were £712 higher for the fast-track group (Table 10). The difference after adjusting for baseline was £799, and this was not statistically significant (95 % CI, -£237 to £1904). Two of the six cases with inpatient stays in the fast-track group had stays substantially greater than for other admitted patients across both trial arms (n = 4 other admitted patients in each arm); further investigation of these two admissions defined them as unrelated to BIS. Excluding these two cases results in costs remaining higher in the fast-track group by £100.

**Table 9:** Service use and costs (2011/12 £s) in two-month period prior to baseline assessment for patients with non-malignant conditions

	Control arm (waiting list) (n = 43)			Intervention arm (fast-track) (n = 44)		
Service	N (%) using service	Mean (SD) contacts-users only	Mean (SD) cost in £s -all sample	N (%) using service	Mean (SD) contacts-users only	Mean (SD) cost in £s -all sample
Inpatient	19 (44)	11.8 (11.5)	2,993 (5,486)	11 (25)	9.7 (7.0)	1,391 (3,112)
Other hospital services	37 (86)	3.5 (4.7)	357 (440)	36 (82)	2.1 (1.4)	236 (204)
GP	36 (84)	2.9 (2.2)	114 (110)	31 (71)	2.4 (1.4)	76 (86)
Nurse	30 (69)	3.8 (2.9)	73 (91)	35 (80)	3.7 (3.8)	91 (134)
Other health services	20 (47)	1.5 (1.0)	27 (68)	18 (41)	1.8 (0.9)	38 (70)
Social and other care	6 (14)	20.0 (28.8)	66 (280)	11 (25)	17.5 (21.1)	119 (303)
Total	3,630 (5,588)			1,952 (3,290)

**Table 10:** Service use and costs (2011/12 £s) in four-week follow-up period between baseline (t1) and t3 for patients with non-malignant conditions

	Control arm (waiting list) (n = 38)			Intervention arm (fast-track) (n = 41)		
Service	N (%) using service	Mean (SD) contacts-users only	Mean (SD) cost in £s -all sample	N (%) using service	Mean (SD) contacts-users only	Mean (SD) cost in £s -all sample
BIS intervention	2 (5)	1.5 (0.7)	4 (19)	3 (95)	2.1 (1.0)	156 (80)
Inpatient^a^	4 (11)	6.0 (3.4)	361 (1,200)	6 (15)	11.5 (8.3)	963 (2,895)
Other hospital services	19 (50)	2.5 (3.5)	145 (262)	20 (49)	1.7 (1.0)	108 (144)
GP	24 (63)	1.6 (0.7)	50 (63)	25 (61)	1.8 (1.2)	49 (57)
Nurse	16 (42)	2.5 (2.5)	28 (62)	21 (51)	2.7 (3.3)	41 (95)
Other health services	4 (11)	1.0 (0.0)	3 (11)	14 (34)	1.5 (1.1)	25 (59)
Social and other care	9 (24)	11.3 (22.8)	68 (269)	8 (20)	5.4 (4.6)	29 (75)
Total	659 (1253)			1,371 (2,948)		

^a^Two of the six cases with inpatient stays in the fast-track group had stays substantially greater than for other admitted patients across both trial arms: these two admissions were unrelated to BIS

The EQ-5D utility scores for the fast-track group were 0.49, 0.58 and 0.59 at baseline, two-week follow-up and four-week follow-up respectively. The figures for the waiting-list group were 0.55, 0.58 and 0.54. The maximum QALY gain over the four-week follow-up period was 0.077. The QALY gain for the fast-track group was 0.0431, while for the waiting-list group it was 0.0430, indicating virtually no difference. This was mainly due to the lower baseline utility score (which is used in the QALY calculations) for the fast-track group. After controlling for baseline EQ-5D scores, it was shown that the fast-track group gained 0.003 extra QALYs (95 % CI, –0.001 to 0.007). The incremental cost-effectiveness ratio indicated that intervention resulted in a cost per QALY of £266,333, which is substantially above the threshold used by NICE (£20,000–£30,000). If the cost difference of £100 (excluding the intervention patients with extreme inpatient costs) is used, the cost per QALY is much less at £33,333 but still above the threshold. The cost-effectiveness plane revealed that there was an 86.5 % likelihood that the intervention had higher costs and resulted in more QALYs. There was only around a 7 % likelihood of lower costs and more QALYs. However, when the two outliers were removed, there was a 54.7 % likelihood of higher costs and more QALYs and a 33.9 % likelihood of lower costs and more QALYs.

## Discussion

In this Phase III RCT of BIS for patients with non-malignant conditions there was the same positive direction of quantitative effect as reported previously for patients with cancer, but the effect was smaller and was not statistically significant; for those with cancer the service had a reasonably sized and statistically significant clinical effect [21]. The health economic findings showed that the intervention increased costs slightly; the costs per QALY were above the thresholds used by NICE. A remarkably similar proportion of patients with non-malignant disease and their carers reported qualitatively that BIS had made a positive difference: 92 % of patients with non-malignant disease and their carers compared with 94 % of those with cancer [21]. For patients with non-malignant disease and their carers, these impacts, the identifiable interventions they found helpful, and the mechanisms of impact were the same as those identified by patients with cancer and their carers. In addition, patients with non-malignant conditions and their carers particularly valued praise of their established strategies and the provision of reassurance, potentially reflecting the longevity of their experience of breathlessness. Thus the result of the analysis of qualitative data from the two sub-protocols was strikingly similar: BIS had made a positive impact for the majority of patients and carers living with breathlessness. Another service using similar complex, predominantly non-pharmacological, interventions has reported a clinically and cost-effective impact in this group [33].

We have considered three areas for discussion which may illuminate reasons for the differences in our main findings between the two disease groups: (1) baseline differences between those living with non-malignant conditions compared to those with cancer; (2) the notable trend of improvement over both trial arms for patients with non-malignant conditions to the key measurement point; and (3) exploration of the difference in magnitude of the quantitative and qualitative results in this mixed method trial.

### Baseline differences between non-malignant conditions and cancer

There were noteworthy clinical differences at baseline between both patients and carers on the sub-protocol for non-malignant conditions (Tables 1 and 2) and the sub-protocol for cancer (equivalent Table 1 in Farquhar et al., 2014 [21]). Although the mean Charlson Co-Morbidity Index was worse (higher) for patients with cancer, the mean Australia-modified Karnofsky Performance Scale score was worse (lower) for patients with non-malignant disease. Similarly mean modified Borg scales and NRS scores for breathlessness, CRQ scores, and both patient and carer HADS scores for anxiety and depression were consistently worse (higher) for those living with non-malignant disease. Importantly, it is worth noting that initial ‘distress due to breathlessness’ (primary outcome measure) was also higher for patients with non-malignant disease and their carers.

Brief reports of work by Chowienczyk et al. [34] and Javadzadeh et al. [35], comparing patients with advanced COPD recruited on the protocol for non-malignant conditions to those recruited on the protocol for cancer, identified statistical and clinical differences between these two disease groups in terms of their Descriptors of Breathlessness and CRQ scores, suggesting that the groups were different in terms of both their experience of, and impact of, breathlessness. Different combinations of clusters of Descriptors of Breathlessness were associated with each diagnostic group; the cluster ‘chest tightness’ was associated with cancer patients [34]. Patients with advanced COPD scored lower across all four CRQ domains than patients with advanced cancer; this was statistically significant for the dyspnoea, mastery, and emotional functioning scores (*p* < 0.05), and clinically significant for the latter two, suggesting poorer respiratory health-related quality of life and a potential difference in referral threshold with a higher threshold for non-malignant disease [35].

### Trend of improvement across trial arms

There was a notable trend of improvement over both trial arms for patients with non-malignant conditions to the key measurement point; that is, there was a clinically significant 1.56-reduction for the control arm by t3 (Table 3). This contrasts with the findings for patients with cancer, who showed very little change by t3 for the control arm on the primary outcome (0.23-reduction) (equivalent Table 2 in Farquhar et al., 2014 [21]). Three potential explanations for this improvement in patients with non-malignant conditions whilst they were in the control condition include regression to the mean, a referral effect, or a therapeutic effect from the research interviews. Regression to the mean is a well-established phenomenon, but less has been written about potential referral effects or therapeutic effects of research interviews.

Considering a potential referral effect, our pre-clinical findings had previously highlighted the isolation experienced by patients with breathlessness and the valued but sporadic nature of existing service contacts [16]. Similarly, Gysels and Higginson have described the “invisibility of breathlessness” in COPD and the impact of this on service access [12]. The fact that a referral to a specialist service such as BIS had occurred could have affected our measured outcomes if this diagnostic group of patients and carers had a sense of anticipation of expert help.

The potential therapeutic effect of research interviews is also worth considering. Similar in effect to measurement reactivity [36] or assessment reactivity [37] (in that this would be an unintended consequence of the data collection process), a therapeutic effect could arise from research interviews where qualitative interviewing techniques provide study participants with the opportunity and time to tell their story, and in response the interviewer actively listens and responds with empathy and understanding. There are neurophysiological explanations for why empathy is helpful in the management of breathlessness [38]. This therapeutic effect was a recurring theme in our qualitative data from patients with non-malignant conditions and their carers, some of whom explicitly stated that talking to their researcher was helpful. A component of the BIS model, and a palliative care approach, is actively listening to patients and carers. Indeed one of the many aspects of the intervention valued by patients was the opportunity to talk about breathlessness to someone who actively listened, and another was having their breathlessness validated or legitimised — our research interviews did both. It is also worth noting  that all five researchers across the two sub-protocols came from either a health or psychology background (three from nursing, one from psychology, and one from physiotherapy).

There is a small but growing literature within palliative and end of life care research of the non-harmful, and even positive, effects of research interviews on study participants (for example, [39–41]); however, this literature usually informs debates on the ethics of palliative and end of life care research and relates predominantly to qualitative observational studies. Here we suggest that, whilst qualitative interviews may indeed have a positive impact on study participants, positive impact may actually have a contaminating effect on quantitative research outcomes, diluting any measurable impact of interventions. This is problematic for the design of mixed method randomised studies of interventions with supportive or psychological components and has been noted by others [42]. The collection of baseline data through postal, rather than face-to-face or verbal methods, may have reduced this effect but at the cost of not obtaining qualitative baseline data and at the risk of greater loss to follow-up.

The more frequently verbalised therapeutic effect of the research interviews in the sub-protocol for patients with non-malignant conditions may relate to the fact that this sub-protocol included more researcher contacts than the sub-protocol for patients with cancer. It may also relate to a greater need for a therapeutic alliance [43, 44] in patients with non-malignant conditions and their carers, or the greater investment in psychological support for patients with cancer as part of supportive care, such as Macmillan Cancer Support and Maggie’s Centres. Patients experiencing breathlessness in non-malignant conditions have usually lived with their condition for much longer than those with cancer. Their trajectory of declining functioning and increasing breathlessness is less steep [45, 46], and so they may develop their own practical strategies to enable life with breathlessness. Thus it may be that BIS is still effective for patients with non-malignant conditions but in a different way than for those with cancer. In cancer, where the illness experience in the breathless patient is usually shorter in duration, the slope of decline in functioning and increasing breathlessness is potentially steeper, meaning they have had less time to self-develop strategies to enable life with breathlessness: thus BIS has to teach these. The reassuring role of BIS was certainly more predominant for patients with non-malignant conditions and their carers. As one of the BIS providers said: *“the needs felt different, but the malignant patients had a lot of acute anxiety about what breathlessness was and what it meant, and how it would be in the future because the worsening of their condition was coming quite quickly to them and their condition was going to worsen […] relatively soon, whereas with the non-malignant patients […] didn’t have those acute anxieties, and it was something they’d lived with for a while, and […] they wanted to know more about perhaps exercising, keeping going and being able to do the things they loved and that slow change and the coming to terms with that slow change, whereas the malignant patients it was all about the very acute anxiety and distress of dealing with this condition that was changing quite rapidly”* [BIS Provider 02].

### Differences in the quantitative and qualitative data

There was a difference in magnitude between the quantitative and qualitative findings of this mixed method trial, with a more positive outcome from the qualitative data. This held true across both sub-protocols, but was more notable in the non-malignant disease sub-protocol.

Wagner et al. state that the “challenges inherent in reconciling apparently conflicting findings from qualitative and quantitative approaches […] has the potential to yield benefits that emerge only through the struggle to reconcile discrepant results and may provide a sum that is greater than the individual qualitative and quantitative parts” [47]. Reviewing the BIS RCT mixed method findings through the lens of Moffatt et al.’s six strategies for exploring “discrepant” or “conflicting” findings facilitated this “struggle” [48]:

i) *Treat the methods as fundamentally different* – The BIS RCT quantitative and qualitative methods shared the ultimate purpose of assessing the effectiveness of BIS (although the qualitative methods sought to do more than this and explore mechanisms of action as well), but they did so in a fundamentally different way: through different, but related, questions and approaches. The methods, and their findings, should therefore be considered complementary rather than confirmatory.
ii) *Explore the methodological rigour of each component* – The BIS RCT qualitative interviews were brief, but were focused and data-rich, and were conducted with the same sample (and across the entire sample); however, we have described above the potentially therapeutic effect of these interviews. Researchers were blinded to the key measurement point for the quantitative primary outcome, and study-specific training and monitoring (all data collection, quantitative and qualitative, was audio-recorded) facilitated quantitative data collection fidelity.
iii) *Explore dataset comparability* – A strength of the mixed method BIS RCT was that the two data types were collected contemporaneously, by the same researcher (caseloads were held) and from the same sample.
iv) *Collect further data and make further comparisons* – The progressive nature of patients’ conditions in the BIS RCT, trial design and funding limits prevented the collection of further data, plus this was felt unnecessary given our response to the first of the strategies of Moffat et al.
v) *Explore the process of the intervention* – The possible referral effect and the delivery of the intervention beyond the BIS RCT protocol was such that the full effect of the service may not have been captured at the key measurement point, as discussed above; the primary outcome may have been collected too soon.
vi) *Explore whether the outcomes of the two components match* – As noted above, the quantitative and qualitative components of the BIS RCT addressed different, but related, questions; thus we would not anticipate, or desire, a perfect match. There was some congruence between the qualitative data and the outcome measure for mastery (a CRQ domain), but the qualitative data revealed some specific concepts not measured by the primary or secondary outcomes, such as confidence and knowledge. Consideration should be given as to whether the ’right’ primary outcome and/or outcome measure was used for the trial, and for the non-malignant sub-protocol in particular, and this is discussed in more detail below.

Our use of the same primary outcome measure for both sub-protocols may have been flawed given the differences between the two groups outlined earlier; however, our paper reporting on the sub-protocol for cancer also questioned the appropriateness of this primary outcome given that some patients who deteriorated on the primary outcome measure also qualitatively reported benefits from BIS [21] (a finding repeated in the sub-protocol for non-malignant conditions). The findings of the unpowered Phase II pilot trial showed the same positive trend for the same primary outcome for patients with COPD, and thus did not suggest it was inappropriate [19].

Interestingly, Moffat et al. considered that, in retrospect, a more relevant outcome for their own study would have been ’ability to cope’ — this is an outcome that, with hindsight, might have been more relevant for BIS too. Our paper reporting on the sub-protocol for cancer noted that BIS may have increased coping capacity [21]. Not only are ’coping’ and ’distress’ different conceptually, but ’coping’ can have both positive and negative connotations, whereas ’distress’ is entirely negative; similarly, ’ability to live with/manage breathlessness’ or ’feeling equipped to live with/manage breathlessness’ can be positive or negative. Another alternative would be an outcome based on ’gains’, which is entirely positive (and is meaningful to the NHS).

Consideration should be given to using multiple primary outcomes. Complex interventions have multiple components which are likely to have multiple (positive and negative) effects; it may be misleading, or naïve, to have one primary outcome measure. To get the true impact of a complex intervention we need to move, as proposed by Carr-Hill nearly 25 years ago, beyond the primary outcome measure: “It has long been recognised that outcomes of treatment are multidimensional and complex, and that, to varying degrees, any single index measure of outcome will be inadequate to capture important differences” [49].

BIS is a palliative care service, and there may be different margins of benefit, and a greater spread of benefits, in palliative care complex interventions, with a cumulative effect from the addition of several smaller (quantitative) outcome benefits. It may simply be unrealistic to expect one primary outcome measure to capture it all. An editorial by Richards skillfully applies the “amalgamation of marginal gains” concept first described by Sir Dave Brailsford, team manager of Team GB’s highly successful cycling team [50], to health care [51]. Brailsford’s approach sought marginal gains from single components of the team’s training in order to improve overall performance; as applied by Richards, these gains relate to ’simple’ nursing interventions which individually make only a marginal difference, but in total reduce discomfort and anxiety. As described earlier, patients and carers reported that BIS helpfully provided ’lots of little tips’. There is also the inherent challenge in palliative and end of life care outcome measurement that we expect deterioration [52].

Consideration should also be given to using mixed method primary outcomes. Normand has noted that “faced with the complex and multidimensional objectives of palliative care, analysis of outcomes and cost-effectiveness needs to embrace the complexity, and […] draw on a range of evidence, complex measurement tools, and a good understanding of context” [53]. As Cawley et al. suggest, qualitatively capturing patients’ actual experience of palliative care is a more accurate measure of how and what patients judge as important in their healthcare, but note the challenge lies in “how we convert the very positive experience of individuals into a language of outcome measures” [54]. In the field of breathlessness, Rocker has asked how we can capture those aspects of breathlessness that really matter to patients and their families and asks whether a numeric rating scale change in response to a therapeutic intervention is sufficient, “or does it tell only part of the story?” [55]. Thus our qualitative data (which was rich, consistent and complete) may simply have encompassed more, been more global, capturing several outcomes resulting from several component interventions delivered by BIS, which may explain the difference in magnitude between the quantitative and qualitative datasets.

If both the quantitative and qualitative findings of the sub-protocol for patients with non-malignant conditions had indicated there was no impact from BIS, or a negative impact, then this might have suggested that: referrals to BIS for patients with cancer were more appropriate; that BIS delivered different interventions to the different disease groups (due to different needs) and that these were more or less effective in the different disease groups; that differences in the service model by disease group had an impact (such as the duration of BIS); or that BIS was simply more effective for patients with cancer. However, the difference in the findings between the two disease groups was only in the quantitative data, thus the data, and the arguments considered earlier in this discussion, suggest: a greater therapeutic effect of the research interviews on patients with non-malignant disease (due either to their previously unmet needs, or a dose response to their additional interview); that the primary outcome was more appropriate for patients with cancer either due to the longer term unrelenting nature of breathlessness in non-malignant disease such that distress remains high, or that BIS delivered different (but still important) interventions to patients with non-malignant disease (due to different needs) that didn’t impact on distress; or that our reliance on a single quantitative primary outcome measure is misguided and we should seek ways to compute multiple outcomes relating to components of complex interventions whilst also placing greater emphasis on qualitative outcomes. A combination of these explanations seems likely given accumulating knowledge suggesting the benefits of BIS-type models of care for breathlessness in advanced malignant and non-malignant disease [21, 33, 56].

## Conclusions

In conclusion, BIS has a positive qualitatively identified impact on patients with non-malignant conditions and their carers, and there was the same positive direction of quantitative effect as reported previously for patients with cancer, but the effect was smaller and was not statistically significant when compared to standard care using the quantitative primary outcome of patient distress due to breathlessness. BIS resulted in slightly increased service costs for patients with non-malignant conditions; to see if BIS is cost effective would require a longer follow-up. There were important differences between participants on the sub-protocol for non-malignant conditions and that for cancer in terms of their baseline characteristics and the notable trend of improvement over both trial arms for patients with non-malignant conditions to the key measurement point; they may also have experienced a greater therapeutic effect from the research interviews which diluted the impact of the intervention. Trials of palliative care complex interventions should consider using multiple, mixed method, primary outcomes and ensure protocols limit potential contaminating therapeutic effects from the research design.

## Additional file

Additional file 1: CONSORT checklist for trials. (DOC 218 kb)

## Abbreviations

BIS: Breathlessness Intervention Service
CI: confidence interval
COPD: chronic obstructive pulmonary disease
CRQ: Chronic Respiratory Questionnaire
CSRI: Client Service Receipt Inventory
CUHNFT: Cambridge University Hospitals NHS Foundation Trust
GOLD: Global Initiative for Chronic Obstructive Lung Disease
HADS: Hospital Anxiety and Depression Scale
MRC: Medical Research Council
NHS REC: National Health Service Research Ethics Committee
NICE: National Institute for Clinical Excellence
NRS: numeric rating scale
QALY: quality-adjusted life year
RCT: randomised controlled trial
UK: United Kingdom
